# Active Acoustic Metamaterial Based on Helmholtz Resonators to Absorb Broadband Low-Frequency Noise

**DOI:** 10.3390/ma17040962

**Published:** 2024-02-19

**Authors:** Reza Hedayati, Sandhya P. Lakshmanan

**Affiliations:** Aerospace Structure and Materials (ASM) Department, Faculty of Aerospace Engineering, Delft University of Technology (TU Delft), Kluyverweg 1, 2629 HS Delft, The Netherlands

**Keywords:** acoustic metamaterial, additive manufacturing, Helmholtz resonator, noise attenuation

## Abstract

The aim of the present work is to design active acoustic metamaterial consisting of an array of Helmholtz resonators and fabricating them using an additive manufacturing technique in order to assist in a reduction in noise levels in aerospace applications. To this aim, initially, a passive metamaterial consisting of an array of 64 Helmholtz resonator unit cells is designed and tested to establish the effectiveness and region of performance. The selected design variable for change is identified as the resonator cavity depth through the frequency response for each parameter of the Helmholtz resonance equation and randomized to achieve a broadband frequency range of the passive metamaterial. An active model of this design (actuated by a stepper motor) is fabricated and tested. The metamaterials are tested under two acoustic set-ups: a closed system aimed at recreating the environment of a soundproof room and an open-system aimed to recreate the condition of an active liner. For the case of passive system, the metamaterial gave sound attenuation of 18 dB (for f = 150 Hz) in open system configuration and 33 dB (f = 350 Hz) in closed system configuration. The attenuation obtained for the active model was 10–15 dB over the mean line performance for the case of closed system and 15–20 dB for the case of open system. The closed system was also tested for performance at multiple cavity depths by setting two wall depths at 10 mm and three walls at 50 mm. This test yielded an attenuation of 15 dB at 180 Hz, the frequency corresponding to 50 mm cavity depth, and 10 dB at 515 Hz, corresponding to 10 mm cavity depth.

## 1. Introduction

A metamaterial is an artificially engineered material built up of unit cell entities that have physical properties that are beyond those of the constituent material itself [1,2]. Even though there is no exact definition for this class of materials, they exhibit unique physical properties on a macroscopic level, such as negative permittivity [3,4], negative magnetic permeability [5], negative Poisson’s ratio [6,7,8,9], negative bulk modulus [10], negative density [10], etc. [11,12,13]. Acoustic metamaterials exhibit the property of controlling and manipulating sound waves in different mediums using their structural arrangements [14]. Based on the design of their structure, acoustic metamaterials can be of different types namely, membrane type [15,16,17,18], plate type [19,20], space coiling [21,22,23,24,25], etc. [26,27]. Tunability of resonance frequency and band gap are crucial factors that cannot be achieved by passive metamaterials. Recently, several researchers have implemented different actuation techniques to yield tunable acoustic metamaterials without requiring change structural modification. Such efforts include arrays of active membrane structures [28,29,30,31], phononic plates [32,33,34,35], negative capacitance shunts [36], Helmholtz resonators or split hollow spheres (SHSs) [37,38,39,40,41], fluid cavities separated by piezoelectric boundaries [42], permeable metamaterials [43,44], origami-based acoustic metamaterial [45], and vibroacoustic metamaterials [46,47].

Due to its relatively simplistic design yet effective working performance, studies are being conducted on incorporating Helmholtz resonators in metamaterials for effective noise attenuation. In the metamaterial based on Helmholtz resonators, the depth per wavelength of each resonator in the array for each targeted frequency can vary, in order to exhibit sub-wavelength resonator units. In cases where the dimensions of the acoustic unit cell structures are fixed, we obtain a passive system working for a pre-set range of frequencies, while in cases where the dimensions of the unit cells are tunable, we obtain an active system in which the range of frequencies can be adjusted. 

**Figure 1:**
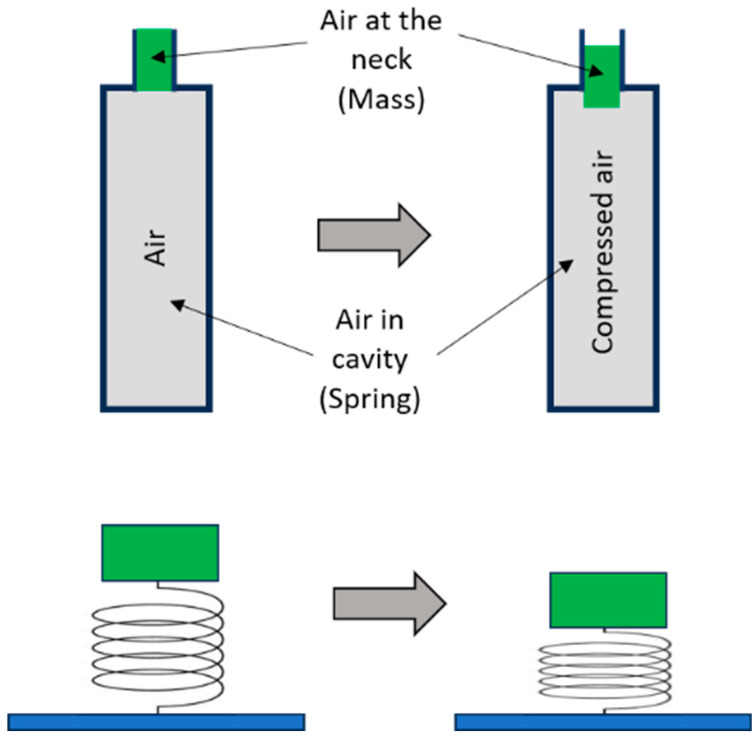
Phenomenon of Helmholtz resonance where air inside the cavity is modelled as a spring mass system (redrawn from [48]).

Helmholtz resonance is a phenomenon where resonance is created when air enters an open hole or a neck that leads into a bigger cavity. In principle, a Helmholtz resonator acts like a mass-spring system with the air inside the neck representing the mass and the air inside the cavity working as a spring. As shown in Figure 1, when air is blown over the top of a Helmholtz resonator, the air at the neck of the cavity is compressed. This leads to an increase in the pressure inside the cavity, which tends to restore it to the original volume. When this process almost comes to an equilibrium state with a restoring force F=−kΔx, the air is pushed from the inside to the outside of the cavity by a certain momentum. This leads to a decrease in the pressure of air inside the cavity, which results in the air being sucked back inside. This phenomenon keeps repeating, thereby making the air inside springy, thus vibrating the mass at the neck of the cavity [48]. 

The above explained concept of Helmholtz resonance can be mathematically expressed by the Helmholtz resonator equation:

(1) $$f=\frac{c}{2\pi }\sqrt{\frac{A}{VL}}$$

where c is the speed of sound, A is the cross-sectional area of the neck, V is the volume of air in the container, and L is the length of the neck. 

Passive structures based on the Helmholtz resonator principle have been designed and studied theoretically and experimentally by Wu et al. [49], Zhao et al. [50], Anwar et al. [51], Ozar et al. [52], etc., aimed at reducing noise transmission levels. As for active acoustic metamaterial based on the Helmholtz resonator, several works have been performed in the last two decades [53,54,55,56,57,58,59,60,61,62]. Different types of actuation mechanisms used for altering different dimensional parameters in active Helmholtz resonators are listed in Table A1 in the Appendix A accompanying the paper. 

More recently, active acoustic metamaterials based on Helmholtz resonators have also been introduced and demonstrated exceptional properties. Ding and Zhao [37] presented split hollow spheres (SHSs) as a multi-band acoustic metamaterial. They showed that each cell in an array of SHS units can exert its resonant function in the multi-band medium independently. They also could reach a negative modulus in the frequency range of 900–1500 Hz. Reynolds and Daley [39] could also reach high levels of isolation in a broad frequency range using SHSs. Cheer et al. [38] implemented an array of Helmholtz resonators enhanced by integration of a broadband feedforward control strategy employing embedded loud speakers.

Reviewing the previous works on actively changing the dimensional parameters of the Helmholtz resonators shows that the previous designs provide attenuation for a single instantaneous frequency, and that they have a relatively complex mechanism of actuation and construction. The goal of the present research is to apply the concept of active actuation at the cell level in a lattice structure composed of an array of cells (cavities). This will provide the capability of attenuating multiple instantaneous frequencies. In addition, the active and passive systems are designed such that the possibilities of ease in manufacturing provided by 3D printing are taken advantage of.

The main objective of this research is to design an active acoustic metamaterial to adaptively control noise signals of different frequencies. To achieve this goal, the project is divided into two major phases. The first phase is to design a passive prototype that targets a broadband frequency range. Based on the performance of the passive model and the frequency range defined, an active system with the capability of changing the volume using a stepper motor is developed. 

## 2. Materials and Methods

### 2.1. Metamaterial Wall Design and Fabrication

After an extensive literature study and comparison of some relevant metamaterial designs based on Helmholtz resonators, an active acoustic metamaterial with Helmholtz resonator unit cells was chosen to study the noise attenuation performance. To this end, a passive design was made to benchmark the limitations and performance expectations of the aimed design. 

#### 2.1.1. Passive Metamaterial 

**Figure 2:**
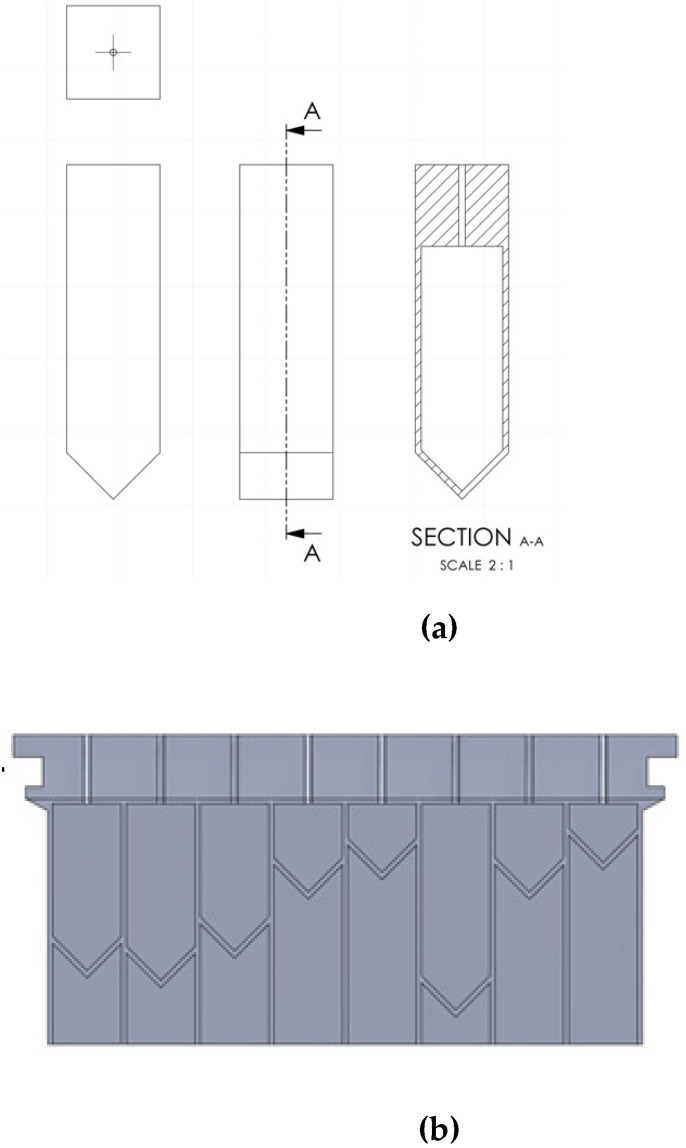
(**a**) The unit cell structure of the passive metamaterial showing the front, top, and section views. (**b**) Cross-sectional side view of the lattice structure of one wall of the passive metamaterial demonstrating the random distribution of cavity depths.

**Figure 3:**
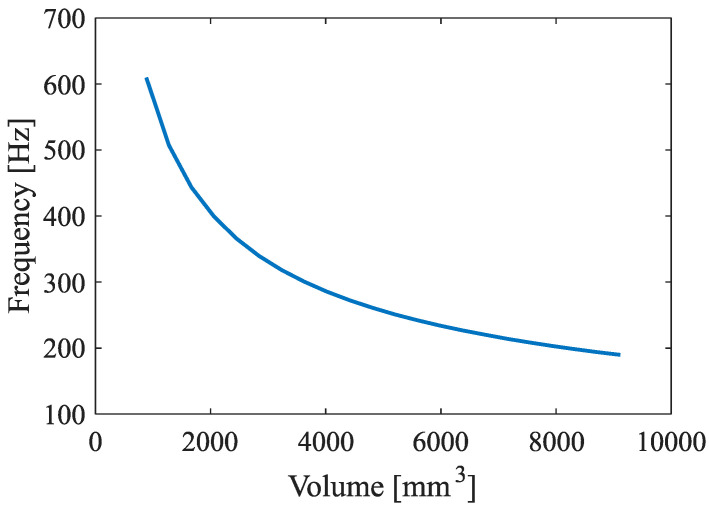
Different cavity volumes and their corresponding frequencies.

To evaluate the metamaterial performance parameters for the final active metamaterial, a passive metamaterial design targeting a fixed range of frequencies is proposed. From the Helmholtz resonance equation (Equation (1)), the impact of each dimensional parameter on the frequency response was investigated, leading to the conclusion that in the lower frequency range, the frequency is highly sensitive to changes in the volume of the resonator cavity. The passive metamaterial was designed to target the frequency range of approximately 150–500 Hz, as this range of frequency is of most significance in the field of noise attenuation in aerospace industries [63,64]. The specific dimensions of each unit cell were derived from the fabrication limitations that set an upper bound to the width of the metamaterial. The constraints (i.e., 3D printer build plate dimension) thus allowed for incorporating an array of 8 × 8 unit cells. The cross-section of the resonator was chosen to be square for the simplicity of fabrication and also because it allows for a large cross-sectional area in each unit cell. The base of the cavity was designed to be V-shaped in order to be able to print the cells without a support structure (Figure 2a). The dimensions of the unit cells have been selected based on the resonance frequency range obtained from the Helmholtz resonance equation (Equation (1)); see Table 1. The dependency of targeted design frequencies with respect to cavity volume is shown in Figure 3. Cavity depths from 8 mm to 42 mm give a volume range of 882–7550 mm^3^ which, according to Figure 3, gives the resonance frequency of 178.6–522.4 Hz.

With reference to the analogy to a mass-spring system (see Figure 1), the effective mass and modulus of the Helmholtz resonator can be obtained. The mass of air inside the neck can be calculated as $m_{n}=\rho AL$. When the air mass in the neck moves downward for distance S due to external pressure, it changes the volume of air inside the cavity for AS. This will increase the pressure inside the cavity for $\Delta P_{c},$ which for an adiabatic transformation (i.e., constant heat) can be calculated as

(2) $$\frac{\Delta P_{c}}{P_{atm}}=-\gamma \frac{AS}{V}$$

where γ is the ratio of specific heat and equals to 1.4 for air. As the net force acting on the mass in the neck is $\Delta P_{c}A$, the Newton’s second law can be written as follows:

(3) $$\Delta P_{c}=\rho AL\;\frac{d^{2}S}{dt^{2}}=KS$$

where K is the spring constant for the system. This gives an equivalent mass and equivalent modulus for the system as follows:

(4) $$m=\rho AL\;K=\frac{\gamma A^{2}P_{atm}}{V}$$

For the range of variable volumes considered for the cavities (8–42 mm), the mass of the system is constant, while the modulus of the system varies between 0.1827 $N/m^{3}$ and 0.048 $N/m^{3}$ for cavity depths of, respectively, 8 mm and 42 mm.

To cover the selected frequency range, the effective volume of the cavity is set by changing the length of the cavity. In the passive design, these lengths are randomly distributed using a randomizer function in MATLAB, over the unit cell array to cover the maximum design frequency range (Figure 2b). 

Fused Deposition Modeling (FDM), a well-known additive manufacturing (AM) technique, was used to manufacture the walls. Polylactic acid (PLA) was chosen as the printing material, and the FDM apparatuses used for this project were Ultimaker 2+ and Ultimaker 3 3D printers. 

#### 2.1.2. Reference Models

**Figure 4:**
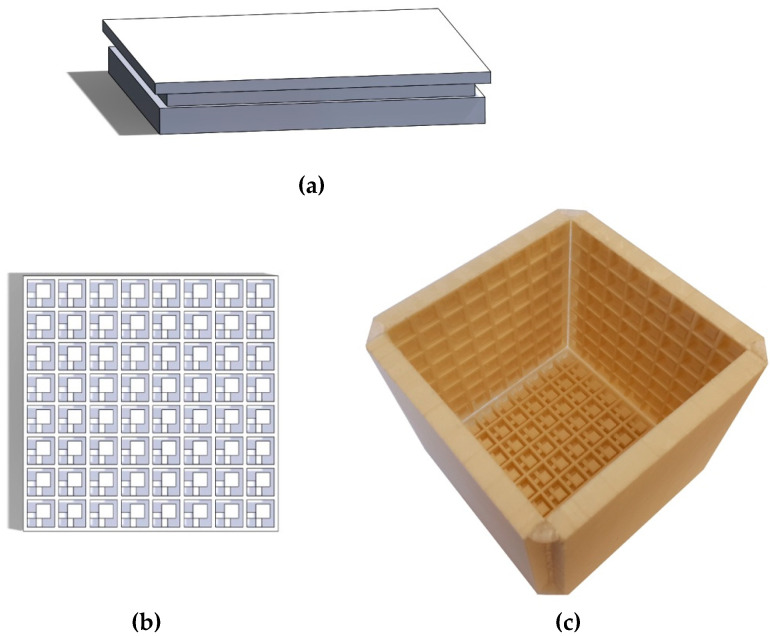
(**a**) Structure of the solid wall of equivalent mass for comparison; (**b**) schematic top view of a resonator wall of the reference acoustic metamaterial; (**c**) assembled metamaterial with 5 walls [65].

To establish a baseline performance comparison for the metamaterial, a solid plate of similar mass (430 g) was manufactured (Figure 4a). As another comparative model, a vibro-acoustic metamaterial developed by Claeys et al. [65] at KU Leuven University was fabricated (Figure 4b,c). The vibro-acoustic metamaterial presented here is a scaled-down model of the one presented in [65], made possible by the 3D printing technique. Some more details on the introduced vibro-acoustic metamaterial can be found in the Appendix A of the paper. 

#### 2.1.3. Electric-Actuated Active Metamaterial

The passive metamaterial presented in Section 3.1.1 targets a range of broadband frequencies of operation. Although this is indeed useful in attenuating noise levels in the range, targeting isolated frequencies across the range is also important. In a passive design, each individual frequency or each sub-range of frequencies that lies within the targeted range of frequencies requires the fabrication of different models that target the required particular or sub-range of frequencies. Since this poses a lot of time and material constraints, an active model was proposed, wherein the volume of the resonators can be altered according to transient frequency requirements. Hence, a single active system can target a range or a set of isolated frequencies, which gives an immense advantage over the passive model. In this design, to vary the volume of each resonator of the metamaterial, the depths of the resonator cavities were varied. As mentioned earlier, the range of change in the depths of the resonator cavities was selected to be from 8 mm to 42 mm, and the variation of depth in the cells of each 8 × 8 array was identical. All other dimensions of the active metamaterial were the same as those of the passive design (Table 1). 

**Figure 5:**
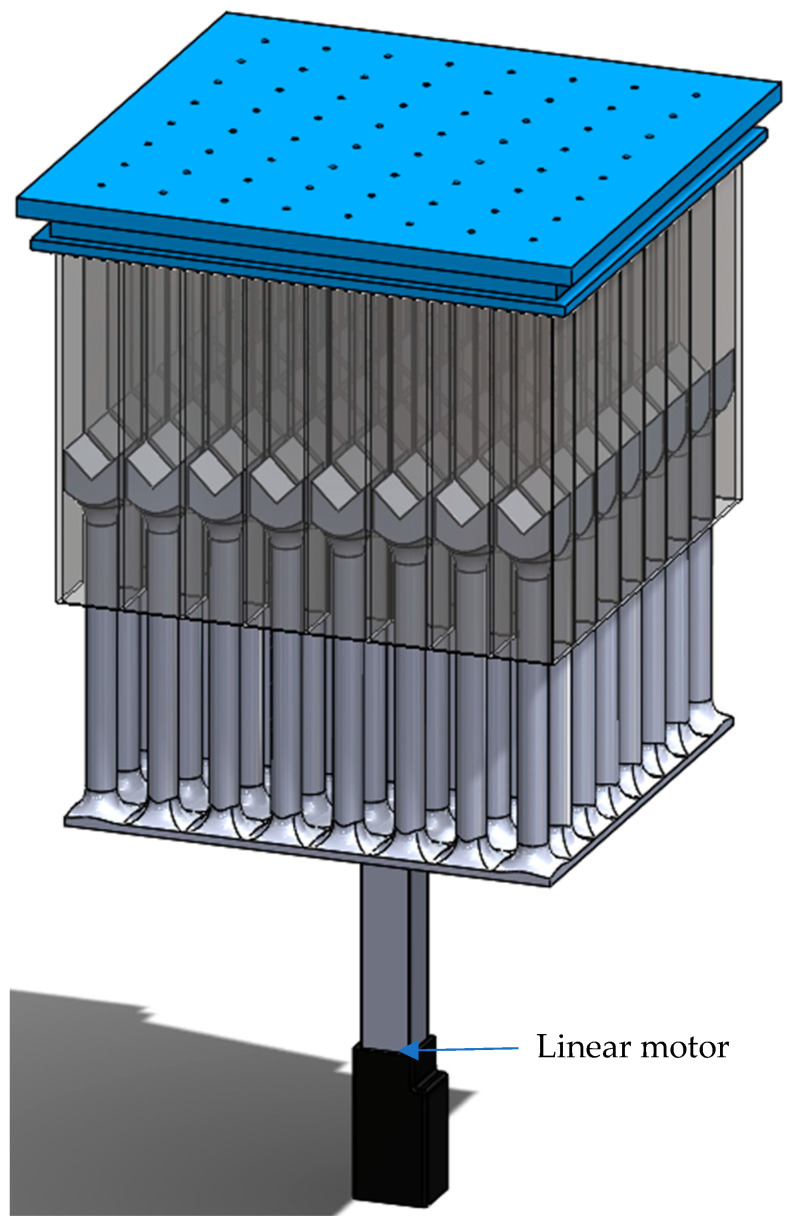
Schematic of the active model containing the cavity bases sliding into the wall cavities by the action of the linear motor attached to the base.

Linear motor actuators, or stepper motors, were used to vary the depth of each wall of the metamaterial. The actuation of different cavity depths was controlled utilizing a potentiometer connected to the control board of a motor. Each linear motor controlled one wall of the metamaterial. A baseplate that supported 64 resonators was connected to the linear motor (Figure 5) to vary the depth of the cavities in accordance with the requirements. To achieve noise attenuation in multiple frequencies, the depths of different walls can be adjusted to different values. One such example for a closed system was performed by setting the depth of two walls at 10 mm and the other three walls at 50 mm. 

### 2.2. Experimental Set-Up

Two configurations were selected for the acoustic measurement: an open system and a closed system. The arrangement was chosen to be cubic in nature for ease of assembly and conformity of both systems. A variety of different designs are possible for the host structure and resonant structures. Since this paper has set out as goal to prove the potential and feasibility of metamaterial-based stop band behavior for acoustic insulation, one possible configuration based on [65] was chosen and analyzed.

**Figure 6:**
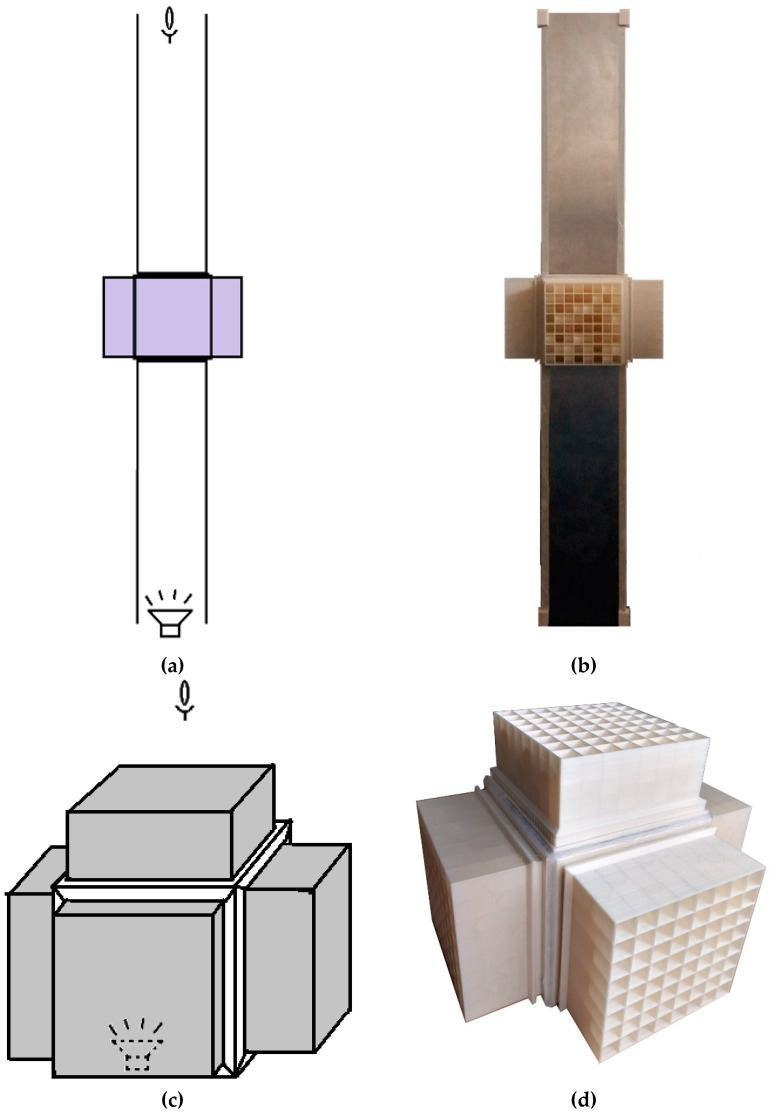
(**a**) Schematic and (**b**) the experimental setup of the open system with the speaker at the bottom and the mic on the top to record the attenuated noise. (**c**) Schematic and (**d**) manufactured closed system with the speaker inside the system and the mic outside to record the attenuated noise.

Helmholtz resonators are widely used in tunnel-shaped structures such as jet engine liners. Different Helmholtz resonator designs have been incorporated into the interior walls of an engine nacelle to mitigate noise generation [40,66]. We designed the open configuration as an extension of the closed system, which can be an indicator of how the Helmholtz resonators would perform in an open channel. In other words, we decided not to change the dimensions, materials, and overall configuration of the walls in the open system for ease of comparison of the performance effectiveness of the proposed metamaterial in enclosed and open applications. The open system was designed to consist of four metamaterial walls arranged in a cubical shape (Figure 6a,b). An aluminum tube was placed on either side of the metamaterial to mimic an open tunnel. A speaker was placed at one end of the tube and a microphone was placed on the other end to measure the noise attenuated after interacting with the metamaterial (Figure 6a).

The closed system was aimed at recreating the environment of a soundproof room. The closed system setup consisted of five walls made of metamaterial structure in a cubical shape with one open side for the speaker such that the five metamaterial walls covered the speaker from all sides (Figure 6c,d). Since the objective of the setup was to seal the noise from the speaker without exiting the space inside the metamaterial, the sound recording device was kept outside the system at a distance of 15 cm from the metamaterial walls. This distance was kept the same for all the tests.

#### Electric Linear Actuation Setup

**Figure 7:**
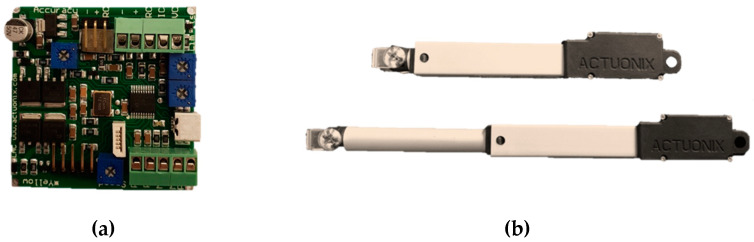
(**a**) Control board and (**b**) linear actuator in its fully contracted and extended states.

**Figure 8:**
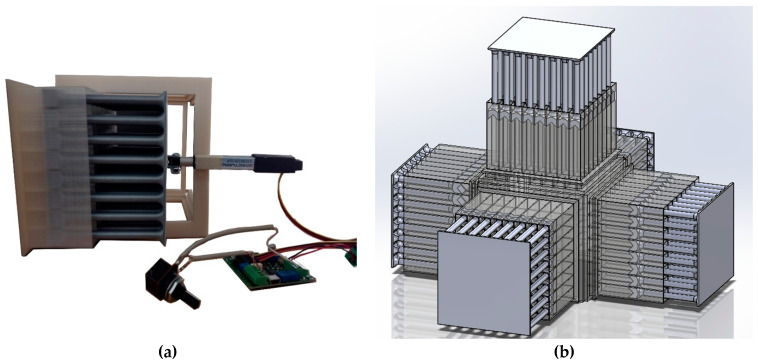
(**a**) Metamaterial wall installed with the linear motor circuit; (**b**) schematic of the five metamaterial walls with five different cavity depths.

A micro linear actuator of model L12-5-210-12-P (Actuonix Motion Devices Inc., Canada), controlled by a potentiometer input was used (Figure 7). To control the position of the actuator head, a control board (Actuonix L.A.C) was used to act as an interface between the potentiometer and the linear motor. The actuator had a precision of about 0.5 mm and a maximum working range of 50 mm. Since the range of the cavity depths was around 40 mm, the actuator was suited to the purpose. Figure 8 shows the linear motor installed onto a metamaterial wall.

### 2.3. Noise Types and Measurement Techniques

Four types of noise samples, namely white noise, pink noise, brown noise, and a frequency sweep of 10,000→20 Hz, were used for the acoustic tests. The noise samples were all acquired from a tone generator application. The difference between the colors of noise used in this study is described below:

- White noise: white noise has a uniform power distribution in any band of a given frequency bandwidth, if the bandwidth is plotted in Hz.
- Pink noise: In homogeny with white noise, the pink noise has a uniform power distribution if the bandwidth is plotted on a logarithmic scale. Consequently, if the frequency spectrum is plotted linearly, the sound amplitude concentrates more on the lower end of the frequency spectrum.
- Brown noise: In brown noise, the power amplitude decreases with a proportion of 1/*f*^2^ with respect to frequency. In other words, in brown noise sample, the power amplitude decreases by 6 dB per octave.
- A frequency sweep of 10,000→20 Hz to provide a wide range of separated frequency noises while measuring loudness in real-time

Additionally, sound samples having isolated frequencies in the range of 100 Hz to 5000 Hz were also tested to evaluate the range of frequencies in which the metamaterials operate.

A Sennheiser e908B cardioid condenser microphone with a frequency response of 40–20,000 Hz was used as the recording instrument. In the results shown for the performance of the active and the passive metamaterials, the sound pressure levels (SPL) were obtained by taking a fast Fourier transform of the recorded sound clips, thus changing the output from a time domain plot to a frequency domain plot. Those plots were normalized with respect to a reference ambient pressure plot. 

## 3. Results and Discussions

### 3.1. Passive Metamaterial

#### 3.1.1. Open System

**Figure 9:**
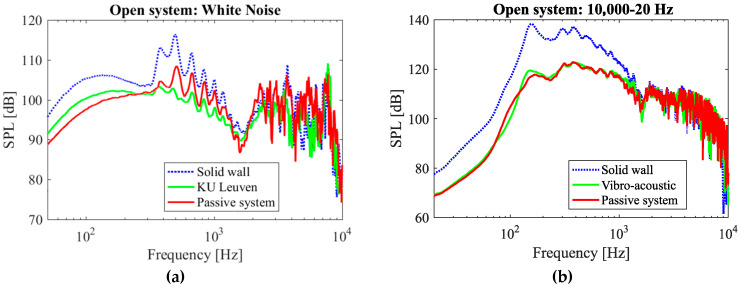

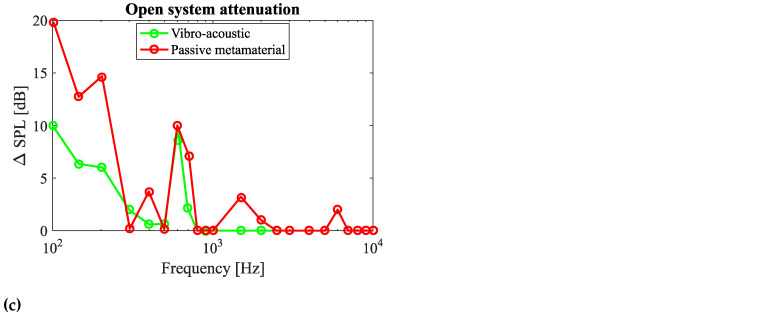
Measured noise amplitude levels in the open system setup for (**a**) white noise sample and (**b**) frequency sweep of 10,000→20 Hz. (**c**) Comparison of attenuation performance of the passive model and the vibro-acoustic model at isolated frequencies.

**Figure A1:**
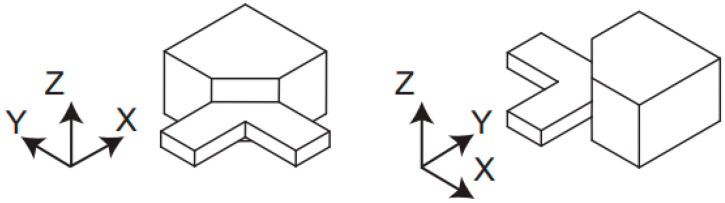
Bending dominant resonant structure in each cell of the vibro-acoustic metamaterial.

In the case of white and frequency sweep 10,000→20 Hz, the open system was capable of attenuating the sound substantially for the designed range of frequencies (up to 550 Hz), and even some sound attenuation was obtained up to about 1000 Hz (Figure 9a,b). The SPL vs. frequency of the system for isolated frequencies is plotted in Figure S1 in the Supplementary Materials. Figure 9c summarizes the attenuation of the passive system at isolated frequencies in a single plot. The passive system shows a substantial attenuation for frequencies up to 700 Hz (Figure 9c). However, the attenuation decays for higher frequencies, as predicted by the designed range of frequencies (Figure 3). As the frequency increases, a sinusoidal wave-like variation in the SPL vs. frequency curves can be observed (Figure A1). This can be attributed to the wavering nature of the frequency output of the speaker unit. This could be confirmed as the same type of fluctuations observed for the case of solid wall (Figure A1). 

From the plots of white, brown, and pink noise (Figure 9a and Figure S1p,q in the Supplementary Materials), as well as the results of isolated frequencies and frequency sweep of 10,000→20 Hz (Figure 9b,c), it can be stated that the metamaterial provides a reasonable attenuation (6–18 dB) for frequencies up to 500 Hz. Besides the optimum performance, the metamaterial also provides a certain degree of attenuation up to 1000 Hz. This is likely due to fabrication inaccuracies, especially because of the changing dimensions of the neck diameter that might have altered the operational frequency range through the design. Finally, a maximum attenuation of around 18 dB at a frequency of approximately 150 Hz can be seen (Figure 9c).

The noise attenuation performance of any material, including mechanical metamaterials, depends on the incoming sound pressure level as well as its frequency. The powers of incoming sound (original dB) for the cases of white noise, pink noise, and isolated frequency tests are not identical at different frequencies. This results in differences in the recorded sound attenuation levels at different frequencies for different sound types.

#### 3.1.2. Closed System

**Figure 10:**
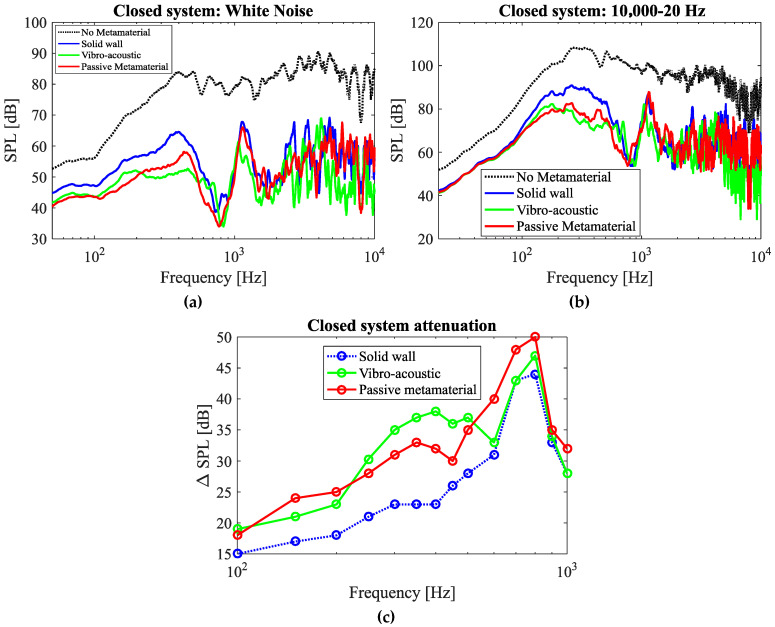
Measured noise amplitude levels in the closed system setup for (**a**) white noise sample and (**b**) frequency sweep of 10,000→20 Hz. (**c**) Comparison of attenuation performance of the passive model, the solid wall, and the vibro-acoustic model at isolated frequencies.

As compared to the open system, the closed system shows a significantly higher attenuation over a larger range of frequencies (Figure 10). Regarding the sound attenuation performance of the solid wall of comparable mass, this can be naturally attributed to the attenuation caused by a solid enclosure. The attenuation beyond the solid wall response is hence taken as the effective result of the presence of metamaterial resonators. It is notable that in the closed system plots, a maximum attenuation of 50 dB is recorded at around 800 Hz (Figure 10c). However, this cannot be considered entirely as a contribution of the passive metamaterial as it is seen that the solid wall model also gives a relatively high attenuation around that frequency. Thus, to identify the effectiveness of the resonators, it would be wise to compare its result to the result of the solid model of the same mass rather than comparing it to the case without metamaterial. By considering this, the maximum attenuation of the passive metamaterial compared to the solid model comes to about 10 dB at 350 Hz (Figure 10c).

The acoustic metamaterials, meanwhile, are not more effective than solid walls at frequencies higher than 1000 Hz (Figure 10); the mere presence of material attenuates noise at higher frequencies. The higher noise attenuation level of the metamaterial at higher frequencies (for instance at 1000 Hz compared to 20 Hz) can be attributed to more contribution of passive noise attenuation mechanism of the bulk material at higher frequencies as well.

In Figure 9 and Figure 10, the performance of the passive system is also compared to that of the vibro-acoustic metamaterial. As the results show, in the case of the open system, the passive metamaterial presented in this research provides much better performance as compared to the vibro-acoustic model. In the case of the closed system, the passive system provides better performance in frequencies lower than 210 Hz. However, in the frequency range of 210–500 Hz, the vibro-acoustic metamaterial provides a better performance after which the passive metamaterial shows a better performance again. 

### 3.2. Electric-Actuated Active Metamaterial

The electromagnetic actuation provides the possibility of altering the dimensions of a large number of resonators in a controlled fashion.

#### 3.2.1. Open System

**Figure 11:**
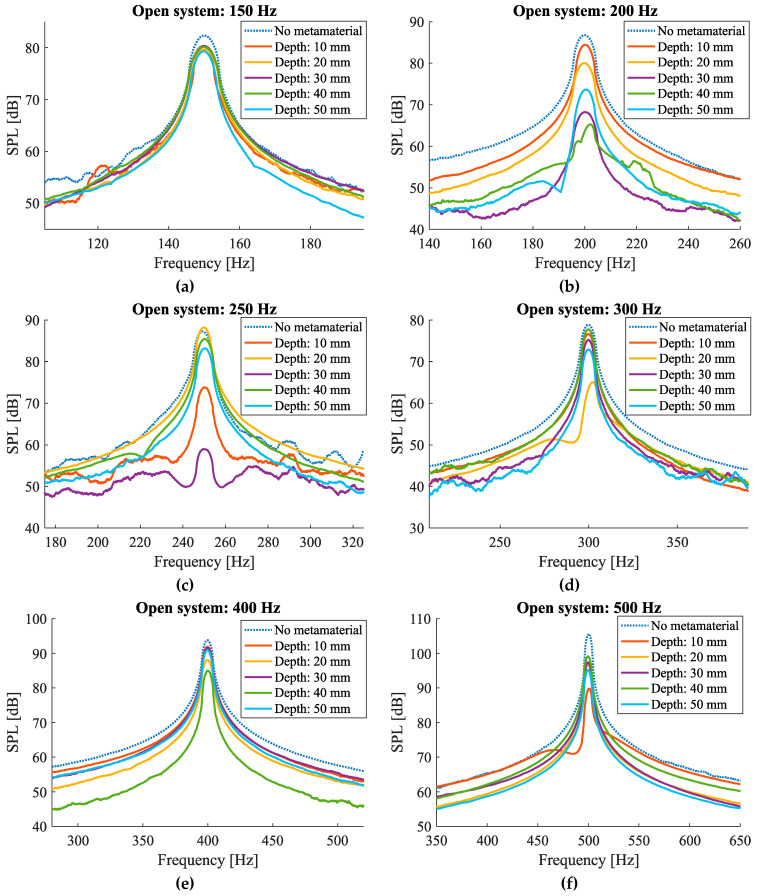

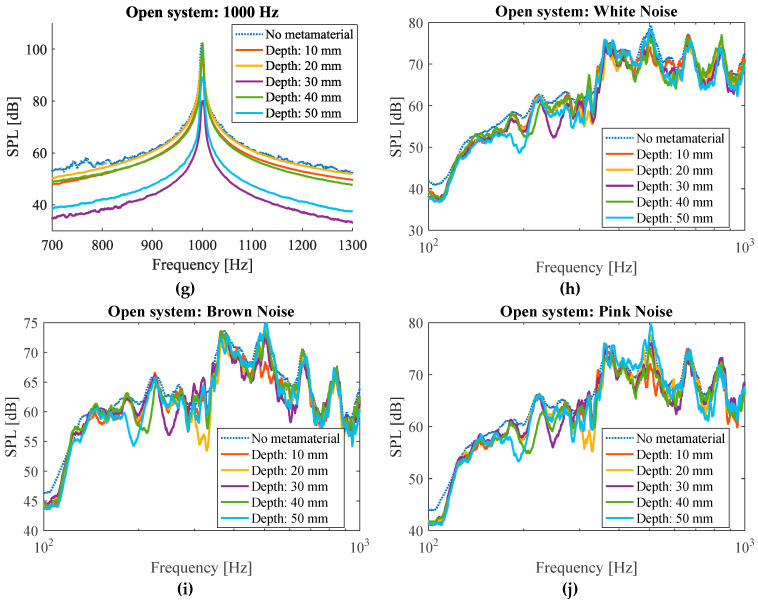
Noise amplitude measurements for the open system for different isolated frequencies as well as white, pink, and brown noise samples.

The results of the open system tests for white, pink, and brown noise samples show that there are reasonably narrow bands of frequencies that are attenuated at the corresponding resonant frequencies for the selected cavity depths (Figure 11h–j). For instance, in the plot obtained for the pink noise sample (Figure 11j), clear dips can be observed at frequencies around 190 Hz, 210 Hz, 235 Hz, 305 Hz, and 500 Hz which, respectively, correspond to 50 mm, 40 mm, 30 mm, 20 mm, and 10 mm cavity depths. Similar frequency dips can be seen in the plots obtained for the white and brown noise samples (Figure 11h,i). 

Such bands also appear in the tests of isolated noise samples, only when the frequency of the noise sample is close to the target frequency of the cavity. For example, the dip corresponding to 50 mm cavity depth with the target frequency of 160 Hz (Figure 3) can be observed in the SPL graph obtained for the isolated frequency of 200 Hz (Figure 11b). Similarly, the dips corresponding to 40, 20, and 10 mm cavity depths with target frequencies of 183 Hz, 272 Hz, and 424 Hz (Figure 3) can be observed in SPL graphs obtained for the isolated frequency of 200 Hz (Figure 11b), 300 Hz (Figure 11d), and 500 Hz (Figure 11f). The dip corresponding to the cavity depth of 30 mm with the target frequency of 215 Hz (Figure 3) could not be observed in any graphs corresponding to isolated frequencies (Figure 11a–g). This implies that since the readings are taken at a 50 Hz interval, if the target frequency of a particular fixed cavity depth lies in between two frequency bands that do not overlap, the maximal effect of the attenuation might not appear in the single frequency measurements before or after the target frequency but are prominently seen in the white noise and the frequency sweep test samples. 

**Figure 12:**
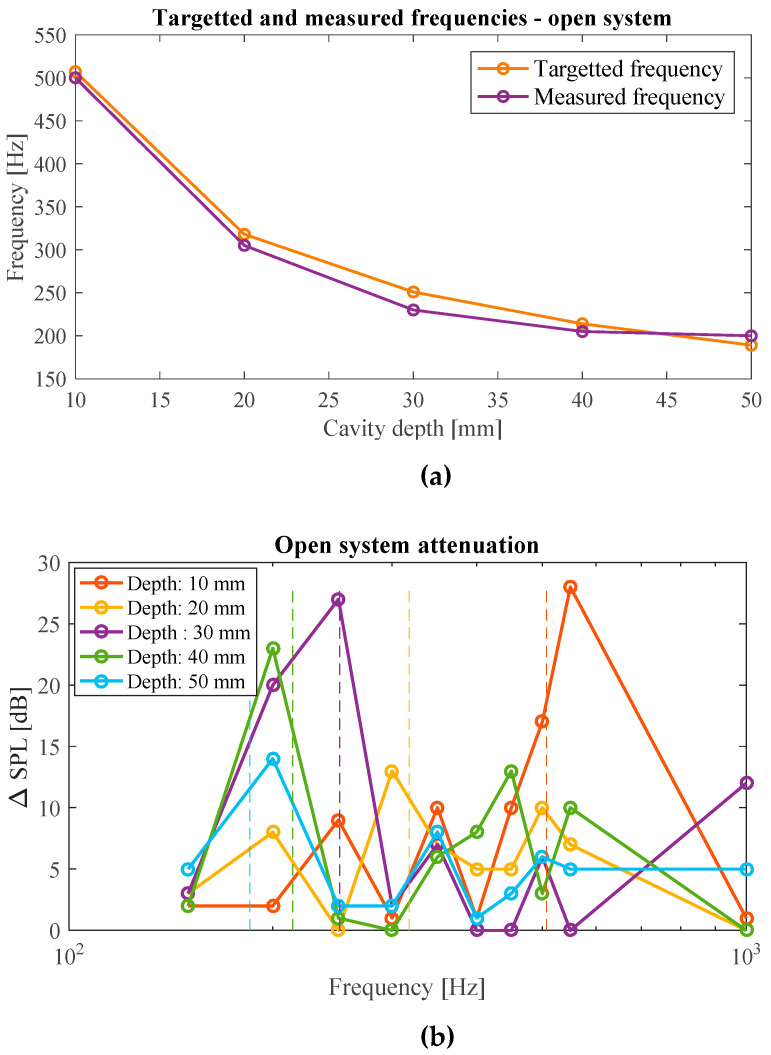
(**a**) Comparison of target and measured frequencies at which the maximum attenuation occurs for white noise sample in the case of open system configuration; (**b**) the measured attenuation magnitude at different frequencies for different cavity heights. Theoretical target frequencies are demonstrated by vertical dashed lines.

The difference between the target frequency and the frequency at which the maximum noise absorption was measured can be seen in Figure 12a for the case of the white noise sample. It can be observed that the theoretical and the measured values have good agreement with each other, and the maximum difference between them is 21 Hz which occurs for the cavity depth of 30 mm. 

Furthermore, the attenuation performances of each cavity depth corresponding to isolated frequency samples are compared in Figure 12b. The vertical lines represent the target frequencies for each corresponding cavity depth, thus specifying the deviation of the peaks from the targeted frequencies. As it can be seen, the maximum attenuation peak of each curve can be seen significantly at the corresponding targeted frequency. For example, the 30 mm cavity depth curve shows maximum attenuation at around 250 Hz, which is also seen in Figure 11c. It can also be noticed from Figure 12b that the magnitude of the attenuation peaks in isolated frequencies are much higher as compared to the attenuations measured for brown, pink, and white noise samples (Figure 11h–j). This behavior of producing a higher level of attenuation while testing for isolated frequencies as compared to large frequency bands is seen to be consistent in all cases tested. 

#### 3.2.2. Closed System

**Figure 13:**
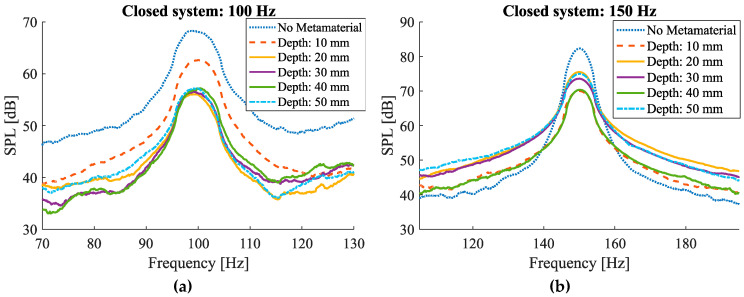

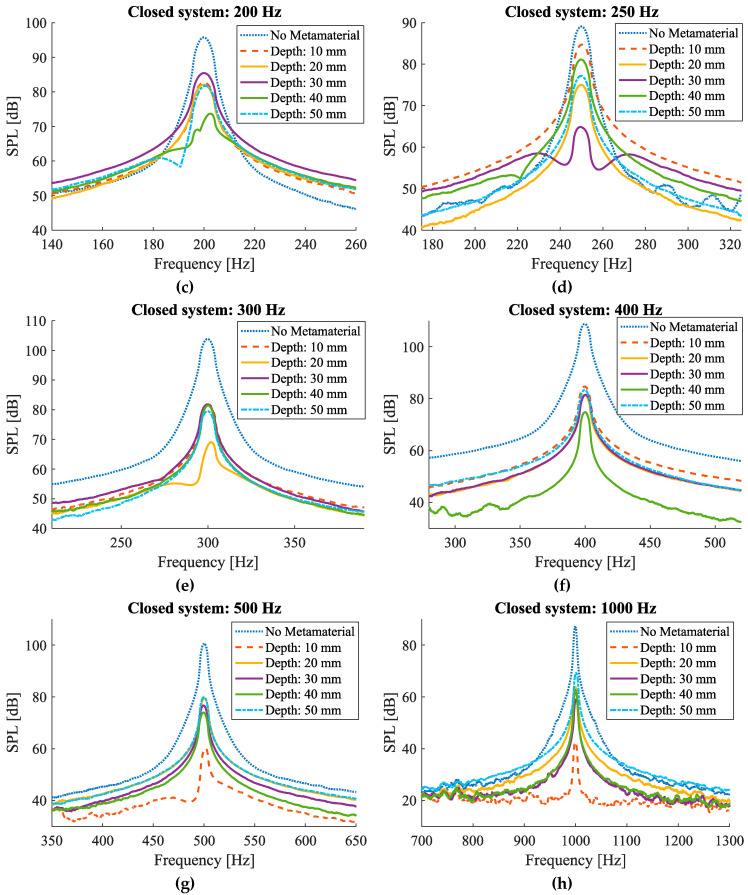

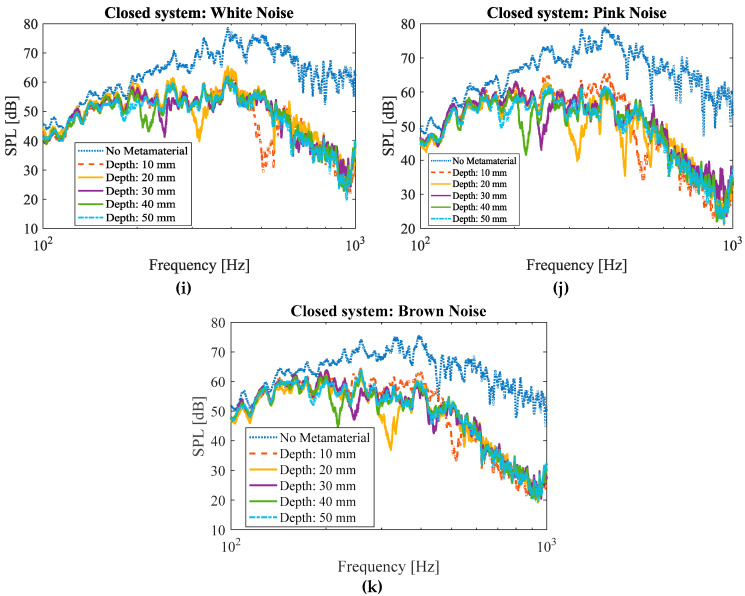
Noise amplitude measurements for the closed system for different isolated frequencies and white, pink and brown noise samples.

The sound attenuation graphs of the closed system case (Figure 13) are comparable to the ones for the open system (Figure 11). As seen in white, pink, and brown noise measurement curves (Figure 13i–k), clear dips in the attenuation levels can be observed at corresponding frequencies. For instance, for the case of brown noise, the frequencies at which the maximum noise attenuation was measured were 500 Hz, 310 Hz, 220 Hz, 205 Hz, and 180 Hz for the cases of 50 mm, 40 mm, 30 mm, 20 mm, and 10 mm, respectively. Similar trends can be seen in the cases of white and pink noise samples. 

**Figure 14:**
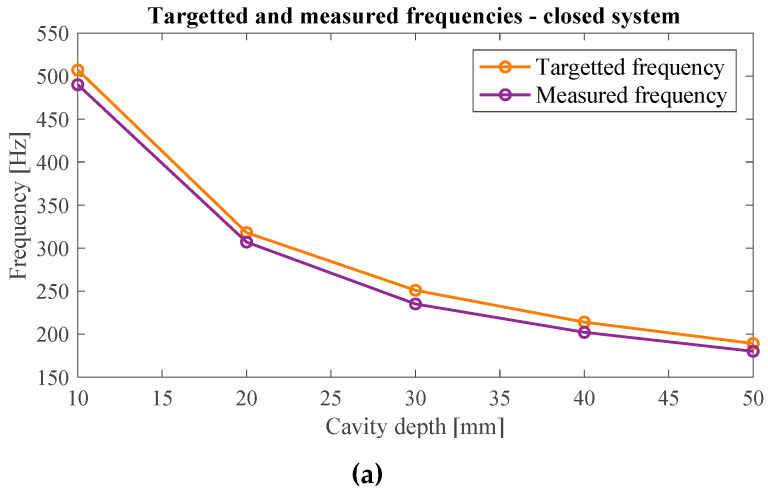

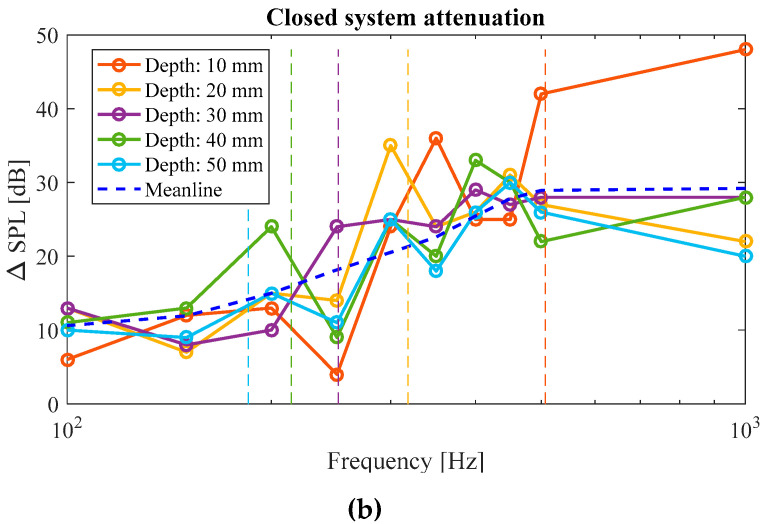
(**a**) Comparison of target and measured frequencies at which the maximum attenuation occurred for white noise sample; (**b**) the measured attenuation magnitude at different frequencies for different cavity heights. Theoretical target frequencies are demonstrated by vertical dashed lines.

Figure 14 compares the target frequencies and the frequencies for which the maximum attenuation is measured for the case of white noise samples. The measured frequencies giving the largest degree of attenuation agree well with the target frequencies. The maximum deviation between the target and obtained frequencies accounts for 17 Hz, which represents a fairly good precision of the metamaterial (Figure 14a). 

The attenuation performance of the closed system measured for the case of isolated frequency noise samples is shown in Figure 14b. The vertical lines represent the target frequencies for each corresponding cavity depth. The performance of the active system was not compared with that of the solid wall since the two systems are of different masses and do not provide a fair metric of comparison. Therefore, the attenuation performance for each cavity depth was compared to the mean line performance of the metamaterial to identify zones of resonator-targeted attenuation. It can be seen that the metamaterial provides an attenuation of up to 15 dB over the mean line performance at frequencies corresponding to each cavity depth. For example, the metamaterial at a cavity depth of 40 mm provides an attenuation of 12 dB over the mean line attenuation of 13 dB at 200 Hz. Similar to the case of open system, this plot also shows that the magnitude of attenuation is greater for the case of isolated frequencies as compared to the attenuations measured for white, pink, and brown noise samples (Figure 13i–k). 

**Figure 15:**
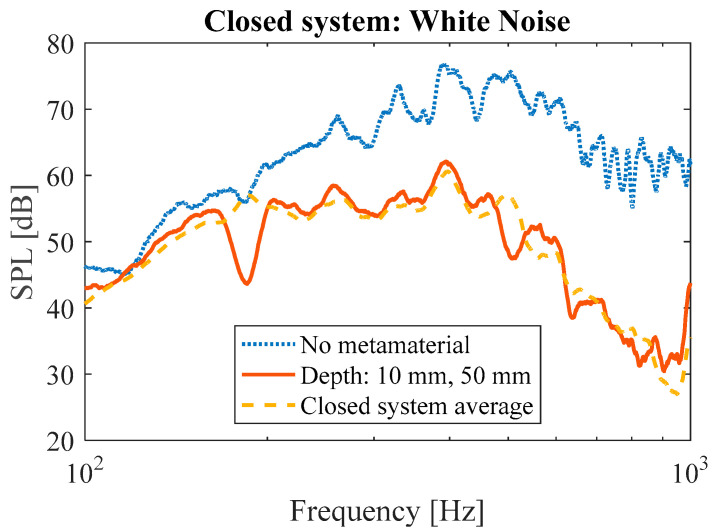
Amplitude measurement showing two simultaneous dips for frequencies at around 190 Hz and 500 Hz.

To demonstrate that the system can be used for different frequencies simultaneously, the depths of the resonator cavities were set to 10 mm for two walls and 50 mm for three other walls of the closed system. The results demonstrate that the metamaterial is capable of targeting multiple frequencies (Figure 15). Clear peaks were observed at 515 Hz and 180 Hz (Figure 15) which are, respectively, close to the target frequencies of 434 Hz (for the cavity depth of 10 mm) and 162 Hz (for the cavity depth of 50 mm). As compared to the average performance of active metamaterial with a single cavity depth, the system with multiple cavity depth yielded an attenuation of 15 dB at 180 Hz (corresponding to the 50 mm cavity depth) and 10 dB at 515 Hz (corresponding to the 10 mm cavity depth).

It must be noted that the electrical system presented in this study is not very space-efficient, as the whole thickness the system requires is at least twice the maximum cavity depth plus the length of the stepper motor (see Figure 8a). By changing the electrical propulsion system to a more compact configuration, a more space-efficient system can be obtained. However, this would not affect the acoustic response of the proposed metamaterial. Another feature that can be added to the proposed active system is a closed-loop control system capable of varying the depth of the cavities as a function of concurrent environmental noise level and dominant frequency.

Table A1 in the Appendix A lists and compares the results of this study with different active Helmholtz resonator designs in the literature used for noise control. Furthermore, the actuation type and the range of dimension change are compared to give an idea of how effectively each type of system affects the output noise. The active system presented in our work was able to attenuate noise in wide bandwidth (100–1000 Hz) with a high level of noise attenuation (up to 50 dB; see Figure 14).

## 4. Conclusions

In this paper, an acoustic metamaterial design based on the Helmholtz resonation principle has been presented, with an active system to target a broadband frequency range, as well as a range of isolated frequencies. A passive model with random cavity depth distribution targeting a low-frequency range (160–434 Hz) was fabricated. The passive model consists of four (for the open system) or five walls (for the closed-system) of an 8 × 8 matrix, of a Helmholtz resonator lattice structure. For the case of the open system, the metamaterial showed its best performance at the frequency of 150 Hz, with an attenuation magnitude of 18 dB. For the case of closed system, on the other hand, the metamaterial showed its best performance at the frequency of 350 Hz, providing a peak attenuation of 33 dB. Based on the performance of the passive model, an active model was fabricated with similar dimensions. The chosen variable parameter for active modulation was again the volume of the resonator cavity through the change in the resonator cavity depth using a stepper motor. The selected depths correspond to different resonant frequencies based on the Helmholtz resonance equation. As expected, the different depths yielded dips in the noise level for the corresponding frequencies, as seen in the plots for the white, pink, and brown noise samples. The system was capable of reducing the noise at a target frequency, with a tolerance of ±20 Hz. At each targeted frequency, the attenuation obtained for the active model was 10–15 dB over the mean line performance for the case of the closed system and 15–25 dB for the case of the open system. The closed system was also tested for performance at multiple cavity depths by setting two wall depths at 10 mm and three walls at 50 mm. As compared to the average performance of active metamaterial with a single cavity depth, the system with multiple cavity depths yielded an attenuation of 15 dB at 180 Hz (the frequency corresponding to the 50 mm cavity depth), and 10 dB at 515 Hz (corresponding to the 10 mm cavity depth). Therefore, the results of this study demonstrated that the tunability of acoustic metamaterials based on Helmholtz resonators and a combination of such systems can effectively attenuate noise in quite a wide low-frequency range (100 Hz to 1000 Hz). The active system presented in this research is a step forward towards the ultimate goal of being able to effectively identify dominant frequencies and reduce their corresponding noise levels through a fully adaptive system that varies more than one parameter to achieve an optimal frequency range with high attenuation capability.

## Figures and Tables

**Table 1:** Dimensions of the resonators and the passive metamaterial walls.

Parameter	Dimensions
Diameter of the neck	1.2 mm
Length of the neck	14 mm
Cross-section of the cavity	14 × 14 mm^2^
Lengths of the cavities	8–42 mm
Area of the metamaterial wall	135 × 135 mm^2^
Depth of the metamaterial wall	61 mm

## Data Availability

All data generated or analyzed during this study are included in this published article (and its supplementary materials).

## Supplementary Materials

The following supporting information can be downloaded at: https://www.mdpi.com/article/10.3390/ma17040962/s1, Figure S1: Measured noise amplitude levels in the open system setup for different noise samples including pink and brown noise, isolated frequencies and frequency sweep from 10,000–20 Hz showing the amount of attenuation achieved for each case. KU Leuven refers to vibro-acoustic metamaterial developed by Claeys et al.

## List of Abbreviations

*A*	Cross-section area of neck
AM	Additive manufacturing
*c*	Speed of sound
*f*	Resonant frequency
FDM	Fused Deposition Modelling
*L*	Length of neck
PLA	Polylactic acid
SPL	Sound pressure level
*V*	Volume of cavity

## Appendix A

### Appendix A.1. More Information on the Vibroacoustic Mechanical Metamaterial

The vibro-acoustic metamaterial model was experimentally tested for different arrangements of the resonator unit cells and was proven to provide significant damping for noise frequencies up to 1000 Hz. Hence, it is expected to provide a proven comparison for the other type of passive metamaterial based on the Helmholtz resonator introduced here. Similar to the Helmholtz resonator, which resembles a mass-spring system with air inside the neck and cavity, the vibroacoustic metamaterial also resembles a mass-spring system with two thin legs resembling the spring and a heavier part resembling the mass (see Figure A1). A low-frequent bending mode creates a resonance in the system. The analogy to mass-spring system allows for controlling the resonance frequency.

### Appendix A.2. Survey on State-of-the-Art Work on Active Actuation

**Table A1:** Comparison of performance and execution of helmholtz resonator based acoustic metamaterials [40].

Type of Volume Change (Neck/Cell)	Decrease in Noise (dB)	Range of Frequency (Hz)	Actuation Type	Range of Changing Parameter	Type (Self-Tuning, Active, Adjustable)	Reference
Length of cavity	Up to 50 dB	100–1000	Electronic-actuated	10–50 mm	Active	This study
Cell Volume change	100%	80–170	Pneumatic	NA	Adjustable	[53]
Neck diameter	4.2 dB	75–115	Rotary-actuated aperture	9–58 mm	Adjustable	[54]
Volume	29 dB	65–150	Rotary-actuated walls	1491 cm^3^–14,093 cm^3^	Self-tuning	[55]
Cavity Length	20 dB	35–46 (Max Attenuation)	Hydraulic	43–243 mm	Active	[56]
Internal Pressure	-	50–500 (Max–160)	Electromagnetic diaphragm/piezoelectric	NA	Active	[57]
Cavity length	18 dB	0–400 (max-226)	-	60–90 mm	Adjustable	[58]
Neck length	-	3–75	Hydraulic	39–60 cm	Adjustable	[59]
Length of cavity	30 dB	80–140	Pneumatic	560–940 cm^3^ 1.8–17 cm	Adjustable	[60]
Area of neck	25 dB	100–3000	Rotary	-	Self-tuning	[61]
Length of cavity	-	100–350	Pneumatic	5–12 cm	Adjustable	[67]
