# Supplementary material for: Active Acoustic Metamaterial Based on Helmholtz Resonators to Absorb Broadband Low-Frequency Noise
Source: Materials (Basel). 2024 Feb 19;17(4):962. doi: 10.3390/ma17040962 (PMC10890402; doi:10.3390/ma17040962)
Supplement: Supplementary file 1 [file materials-17-00962-s001.zip › materials-2764196-supplementary.pdf]

# Supplementary Materials

## S.1. More results

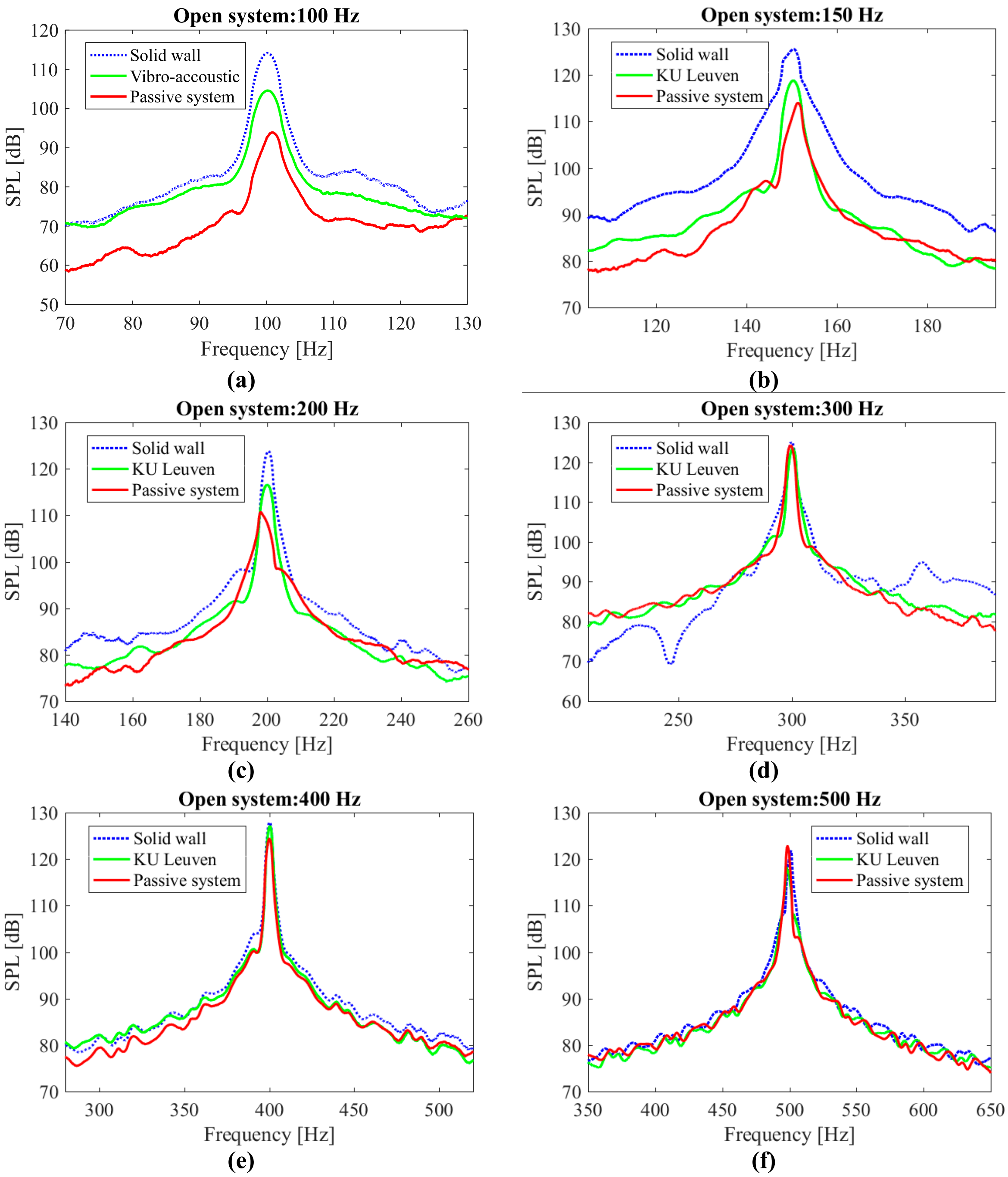

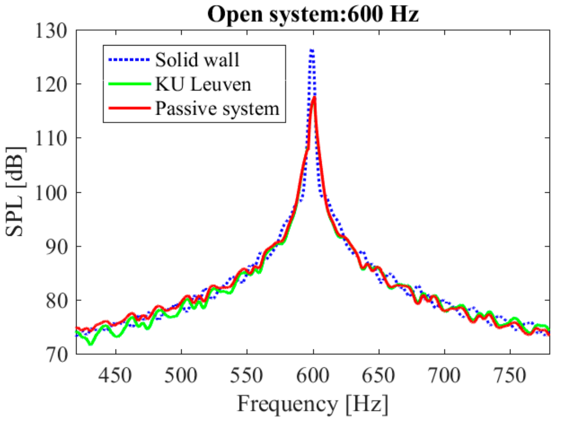
Open system:600 Hz
Solid wall
KU Leuven
Passive system
SPL [dB]
Frequency [Hz]
130
120
110
100
90
80
70
450
500
550
600
650
700
750
(g)

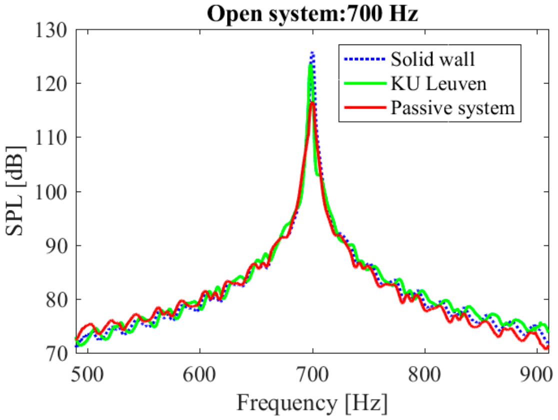
Open system:700 Hz
Solid wall
KU Leuven
Passive system
SPL [dB]
Frequency [Hz]
130
120
110
100
90
80
70
500
600
700
800
900
(h)

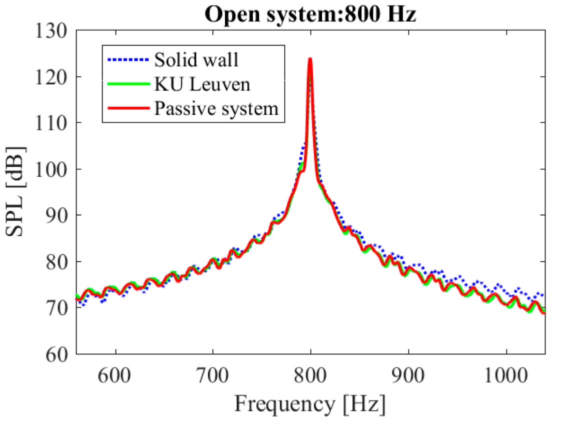
Open system:800 Hz
Solid wall
KU Leuven
Passive system
SPL [dB]
Frequency [Hz]
130
120
110
100
90
80
70
60
600
700
800
900
1000
(i)

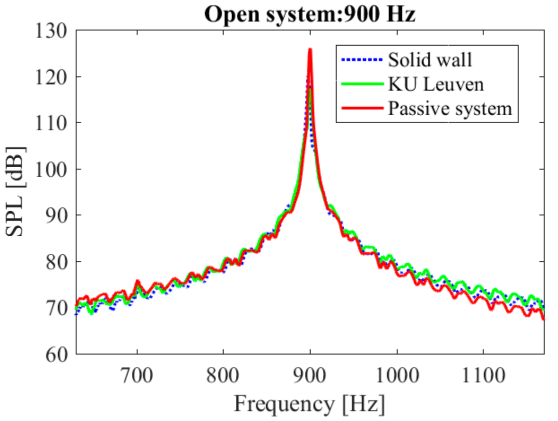
Open system:900 Hz
Solid wall
KU Leuven
Passive system
SPL [dB]
Frequency [Hz]
130
120
110
100
90
80
70
60
700
800
900
1000
1100
(j)

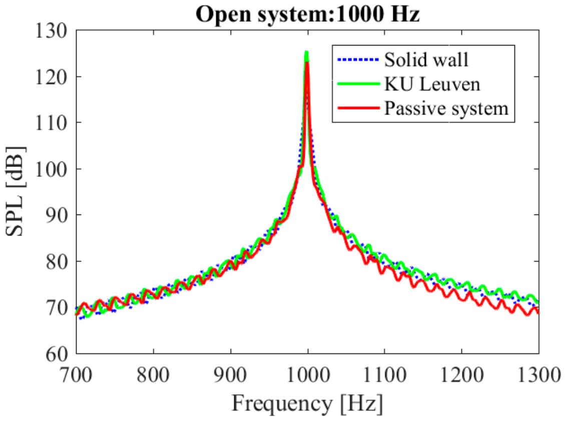
Open system:1000 Hz
Solid wall
KU Leuven
Passive system
SPL [dB]
Frequency [Hz]
130
120
110
100
90
80
70
60
700
800
900
1000
1100
1200
1300
(k)

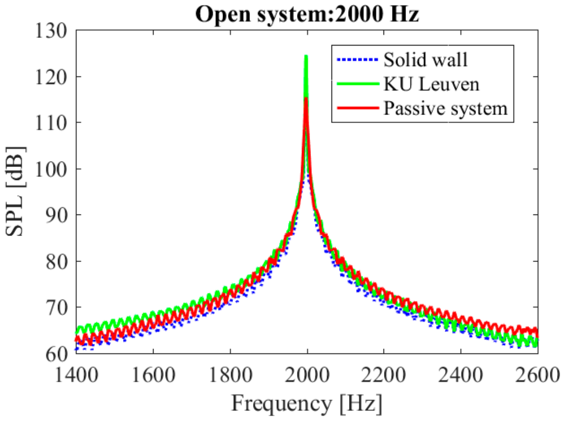
Open system:2000 Hz
Solid wall
KU Leuven
Passive system
SPL [dB]
Frequency [Hz]
130
120
110
100
90
80
70
60
1400
1600
1800
2000
2200
2400
2600
(l)

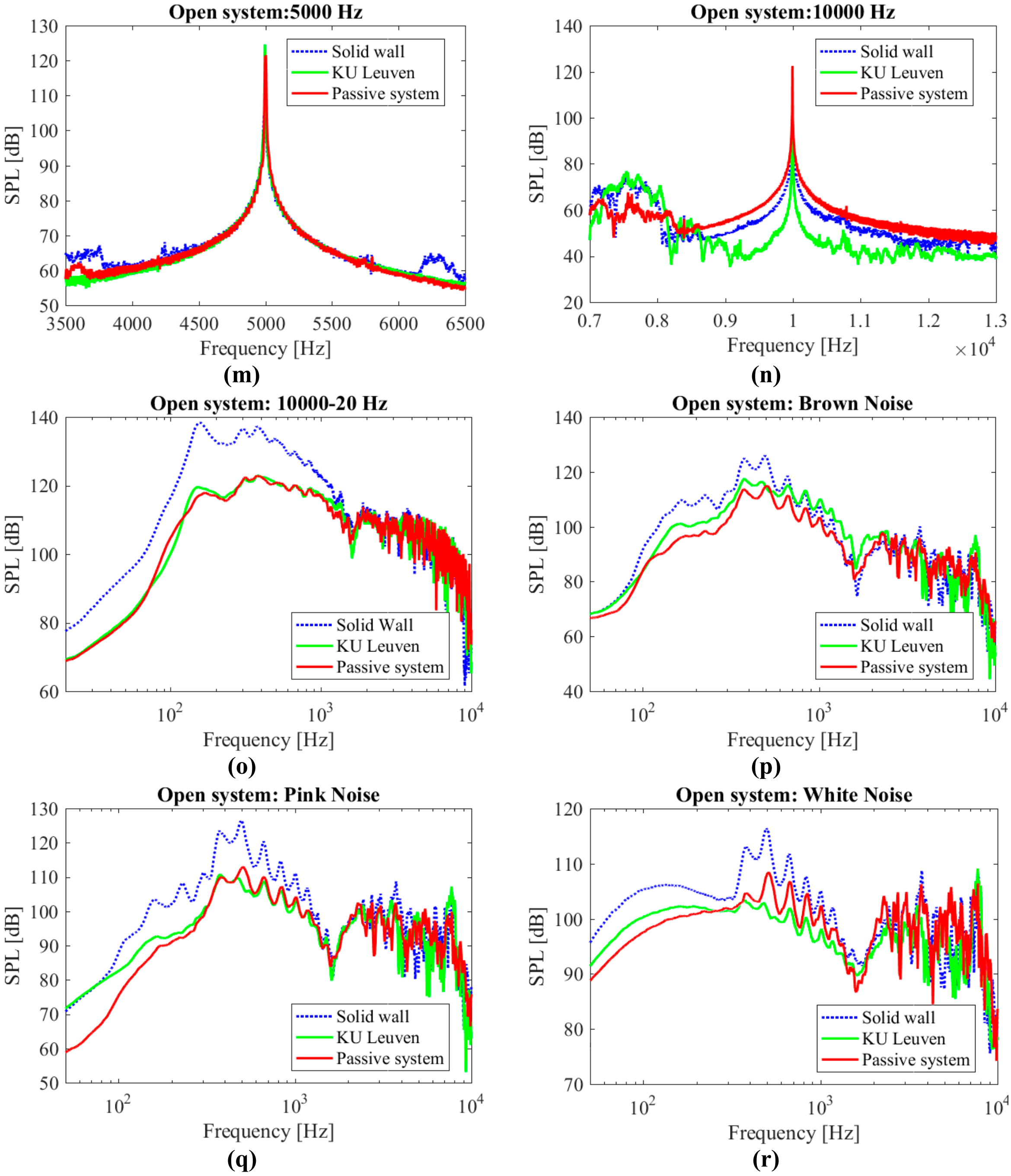


**Figure S1: Measured noise amplitude levels in the open system setup for different noise samples including pink and brown noise, isolated frequencies and frequency sweep from 10000 - 20 Hz showing the amount of attenuation achieved for each case. KU Leuven refers to vibro-acoustic metamaterial developed by Claeys et al. [1]**

# Reference

1. Claeys, C., E. Deckers, B. Pluymers, and W. Desmet, *A lightweight vibro-acoustic metamaterial demonstrator: Numerical and experimental investigation.* Mechanical Systems and Signal Processing, 2016. **70-71**: p. 853-880.
